# Predictive Modeling of Heart Rate from Respiratory Signals at Rest in Young Healthy Humans

**DOI:** 10.3390/e26121083

**Published:** 2024-12-11

**Authors:** Carlos M. Gómez, Vanesa Muñoz, Manuel Muñoz-Caracuel

**Affiliations:** 1 Human Psychobiology Laboratory, Experimental Psychology Department, University of Seville, 41018 Seville, Spain; lmunnoz@us.es (V.M.); manuelmunozcaracuel@outlook.com (M.M.-C.); 2 Hospital Universitario Virgen del Rocio, 41013 Seville, Spain

**Keywords:** heart rate, respiration, predictive modeling, human physiology, simulation

## Abstract

Biological signals such as respiration (RSP) and heart rate (HR) are oscillatory and physiologically coupled, maintaining homeostasis through regulatory mechanisms. This report models the dynamic relationship between RSP and HR in 45 healthy volunteers at rest. Cross-correlation between RSP and HR was computed, along with regression analysis to predict HR from RSP and its first-order time derivative in continuous signals. A simulation model tested the possibility of replicating the RSP–HR relationship. Cross-correlation results showed a time lag in the sub-second range of these signals (849.21 ms ± SD 344.84). The possible modulation of HR by RSP was mediated by the RSP amplitude and its first-order time derivative (in 45 of 45 cases). A simulation of this process allowed us to replicate the physiological relationship between RSP and HR. These results provide support for understanding the dynamic interactions in cardiorespiratory coupling at rest, showing a short time lag between RSP and HR and a modulation of the HR signal by the first-order time derivative of the RSP. This dynamic would optionally be incorporated into dynamic models of resting cardiopulmonary coupling and suggests a mechanism for optimizing respiration in the alveolar system by promoting synchrony between the gases and hemoglobin in the alveolar pulmonary system.

## 1. Introduction

Metabolic and autonomic processes in humans and animals are regulated by control systems that facilitate adaptation and survival in response to internal or external changes. This adaptive process is essential for maintaining stability and is a well-documented phenomenon known as homeostasis, which plays a critical role in maintaining an efficient functional state [1]. Canon [2] introduced the term to describe the feedback operation in physiological systems and the maintenance of equilibrium. It is now recognized as an inherent self-regulatory system within biological systems that is necessary for the state of normal functioning [3].

Biological signals of the nervous system typically exhibit inherent oscillations with varying characteristics, including frequency and temporal variations. The precise variability of these signals is often unclear [4]. Oscillations are influenced by environmental factors and intrinsic processes, including homeostasis and feedback. For instance, spontaneous oscillations in heart rate (HR) have been extensively studied by frequency decomposition, revealing their associations with various activities of the sympathetic and parasympathetic nervous systems [5]. Similar studies have been extended to other biological systems.

HR and respiration signals (RSP) and their oscillations are amplitude-modulated and synchronized [6,7], considering that respiration influences the cardiac response. Inspiration increases HR, while expiration decreases it, a phenomenon known as respiratory sinus arrhythmia (RSA), which is widely accepted as an index of vagal activity enhancing pulmonary gas exchange [7,8]. The physiological system primarily responsible for the interaction between respiration and circulation is the autonomic nervous system (ANS). However, the physiological mechanisms underlying the modulation of cardiac responses by respiration are complex and multifactorial. Both HR and RSP signals are influenced by external and internal mechanisms. Cardiovascular variability is mainly modulated by the two branches of the ANS, which affect the redistribution of blood volume, blood flow, heart rate, and heart variability, but its modulation is also proposed to be largely influenced by breathing. These effects are complex and affect cardiac responses at multiple levels.

The relationship between RSP and HR, often referred to as cardiorespiratory coupling (CRC), has been successfully modeled by complexity measures [9,10,11,12]. These studies focusing on CRC and nonlinear dynamics provide insights into physiological regulation in the context of adaptive mechanisms to stress, aging, and physical training. For instance, stressors such as dehydration have been related to reduced synchronization indices and altered directional relationships between cardiovascular and respiratory variables [9]. Aging further modulates these dynamics, with the resting state showing weakened respiratory contributions to cardiac regulation, while stress conditions amplify these impairments [10]. In contrast, athletes have shown stronger CRC compared to sedentary individuals, reflecting enhanced autonomic synchronization [12]. Specific training interventions, such as inspiratory muscle training, have shown the potential to enhance CRC by promoting central respiratory and autonomic adaptations, with effects observed during active challenges and subtle improvements at rest [11].

The use of linear relationships involving the RSP and its first-time derivative, as a possible causal signal for HR has been explored [13], suggesting an important role for stretch receptors in modulating HR. However, the continuous dynamic relationship between the RSP and HR signals has not been intensively explored. The demonstration of the role of the first-order time derivative of the RSP term in the prediction of HR from RSP would suggest the possibility of linearizing this relationship in the whole RSP cycle during resting state respiration and, given the quasi-sinusoidal character of the RSP signal, it would be possible to model the regulation of the time-lag between RSP and HR by controlling the gains of the RSP and its first-time derivative. Such a model, once empirically validated, would allow to explore by simulation of the HR signal, the regulation of the physiological delay between the RSP and the HR signals, by modulating the amplitude of the signals conveyed to the central nervous system by slow and fast adapting pulmonary stretch receptors [13].

The respiratory rhythm is typically generated by respiratory peacemaker cells in the brainstem, and its oscillations are modulated by central and peripheral reflexes that adapt in response to environmental demands [14]. The origin of the RSA and/or its modulation is currently a matter of debate, several hypotheses could be complementary. In fact, such a complex regulation of the cardiorespiratory coupling would give the system great robustness to maintain an adequate level of function. These proposed mechanisms for the generation and/or regulation of RSA are (i) a central mechanism exerted by the brainstem sympathetic and parasympathetic nuclei, which would be controlled by the central respiratory nuclei through a central pacemaker [15,16,17,18]; (ii) one of the main regulatory nodes of RSA would be in the baroreceptor reflex, as evidence from baroreceptor suction shows its strong influence on RSA, for instance, in forced apnea, RSA is induced by cyclic suction stimulating carotid baroreceptors, suggesting a crucial role for the baroreceptor reflex. Under more natural conditions, the inspiratory phase would increase venous return and then increase arterial pressure, inducing an increase in baroreceptor activity that would activate vagal nuclei in the brainstem [14]; (iii) the activation of pulmonary stretch receptors, generating the Hering-Breuer reflex, which by modifying the central pattern generator of respiration would also modify the vagal response [19]; (iv) but also mechanical pressure exerted over the atrial sinus can modulate the RSA, the so-called Bainbridge reflex [20], and (v) hypercapnia, oxygen pressure, and acidosis acting on central and peripheral chemoreceptors can modulate respiratory sinus arrhythmia [21,22,23]. The controversy regarding the relative importance of central or reflexive mechanisms in the generation and/or modulation of RSA is a long-standing debate with a difficult answer [24] and will not be directly addressed in this report. It is worth noting that both HR and RSP serve the primary purpose of maintaining homeostasis through the balanced and proper functioning of these physiological mechanisms.

In addition to RSA, other types of cardiorespiratory coupling include cardiorespiratory phase synchronization (CRS) and cardiorespiratory coordination (CRCo) [25,26]. CRS refers to the tendency of the heartbeat to align with a specific phase of the respiratory cycle, often observed during deep sleep and under anesthesia. On the other hand, CRCo examines the relationship between the heartbeat and the preceding and following respiratory onset in the time domain. While CRS allows us to analyze how the timing of heartbeats aligns with specific phases of the respiratory cycle [9], CRCo can adapt to variations in respiratory cycle lengths, capturing dynamic changes in the heart–respiration relationship, and some studies have shown their differences in the degree of information provided by the two approaches (relaxation for CRS and stress for CRCo), the CRCo being independent of the length of the respiratory cycle unlike the CRS [26]. These terms could be confused and related to the RSA, but their effects can be observed both independently and in conjunction with RSA. Although there are relevant terms in the understanding of cardiorespiratory coupling, the present study does not attempt to integrate them as they are beyond the main objective of the study.

The present report aims to improve the understanding of the predictive heart rate estimation based on respiratory signals. By analyzing this relationship from a statistical and dynamic point of view, this will provide more accurate information for the modeling of this process. Specifically, we explore the possibility of predicting the continuous heart rate cardiac signal from the respiratory, inspiratory, and expiratory signals in healthy young adults during a spontaneous resting state. Furthermore, the present results are intended to highlight some dynamic aspects of the relationships between RSP and cardiac signals, which would be optionally useful for physiologists and modelers to incorporate in functional descriptions of the relationship between RSP and HR in a resting state.

The main objective of the present report is to model the continuous linear dynamic relationship between the RSP and HR signals, incorporating the first-order time derivative of the RSP [13]. This will support the existence of an anticipatory signal of the respiratory state of the lungs (inspiratory inflation and expiratory deflation) which would help to modulate the time-lag between RSP and HR. Obtaining such a relationship would suggest a possible physiological mechanism to optimize gas exchange by better aligning the RSP and HR. This could possibly be supported by pulmonary fast and slow adapting stretch receptors and/or baroreceptors and chemoreceptors, which would modulate the vagal response and then HR. It can be hypothesized that the continuous HR signal can be predicted from the continuous RSP signal and its first-time derivative, and as a consequence, modulating the gain of these terms will allow us to adjust the time-lag between the respiratory and cardiac signals.

## 2. Materials and Methods

### 2.1. Participants

A sample of 45 volunteer subjects (18 males and 27 females, mean age = 26.71 ± 4.41 SD) participated in the present study. All of them were healthy subjects without any neurological or psychological disorders. They were university students who reported no cardiopulmonary problems or any other major health issues. They were recruited through advertisements at the University of Seville. Before the study, they were informed about the procedure and then signed an informed consent form. The study was approved by the Bioethical Committee of the Junta de Andalucía (1045-N-21 and 1717-N-22) and followed the rules of the latest revision of the Declaration of Helsinki for human research.

### 2.2. Experimental Session

**Figure 1:**
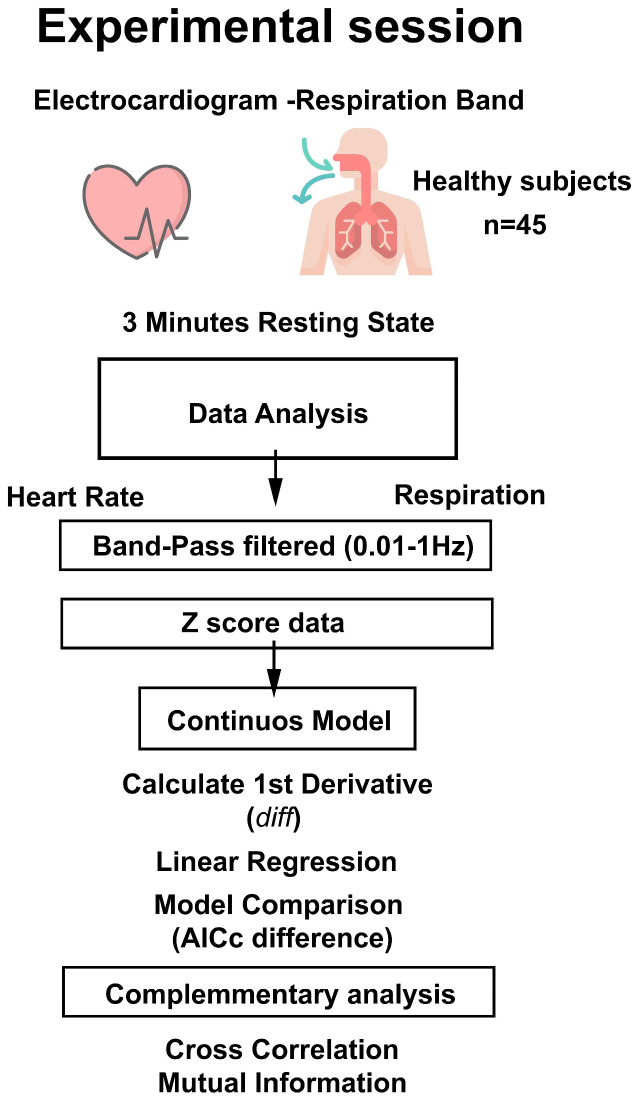
Flowchart of signal processing. Flowchart showing the experimental session and subsequent data analysis. AICc: Akaike information criterion.

Peripheral ECG and RSP signals were recorded using an MP160 (BIOPAC Systems, Goleta, CA, USA) with two amplifier modules (ECG-100C and RSP-100C) (Figure 1). The ECG was collected using three Ag-AgCl lead electrodes (positive, negative, and ground, TSD203 transducer) placed on the left wrist, right wrist, and right ankle, respectively. The RSP signal was recorded by a transducer band placed on the chest (TSD201 transducer). Participants were instructed to move as little as possible and to look at the screen for 3 min without any task or response. Respiration during this period was unassisted and spontaneous. The gain was set to 1000 in the ECG and 10 in the RSP amplifier. The sampling rate was 1000 Hz. Data acquisition was performed with AcqKnowledge v.5.0.1 software (BIOPAC Systems).

### 2.3. Data Analysis

**Figure 2:**
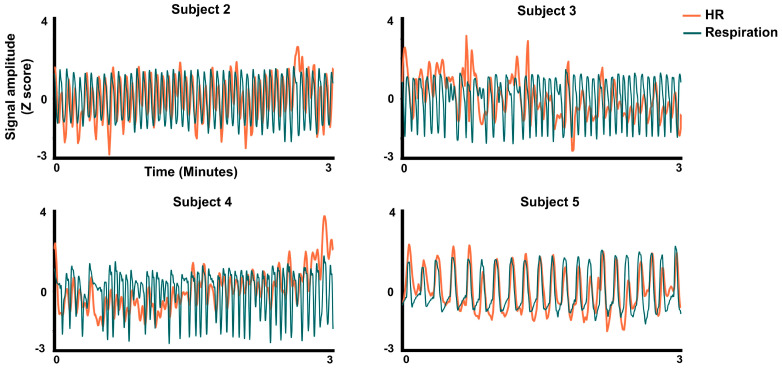
Signal plots. Three minutes recording for the HR and respiratory signals in z-score values for four subjects.

For data analysis (Figure 1), the ECG signal was first processed in AcqKnowledge software, to calculate the HR time series from the ECG signal. The processed signals were exported to Matlab R2019b (MathWorks), where a custom script was used to extract 3 min of spontaneous activity for each subject (Figure 2). To ensure that the analysis captured relevant physiological oscillations while minimizing noise, both HR and RSP signals were band-pass filtered between 0.1 Hz and 1 Hz using a zero-phase filter filtfilt. This range was chosen to include the RSA dynamic, considering that the respiratory rate is around 0.1 to 0.3 Hz, and the HR variability ranges are around 0.04 to 0.4 Hz, and specifically, the RSA effect has been related to the HF component > 0.15 Hz [27]. Neither the RSP nor the HR signal showed clear trends after applying the high-pass filter at 0.01 Hz. The filtered signals were then standardized using z-score.

### 2.4. Signal Analysis

To analyze the possible time-lag delay of the HR signal with respect to the RSP signal, a cross-correlation analysis between the two signals was calculated in Matlab using the xcorr function. In addition to cross-correlation, mutual information (MI) analysis was performed to assess the non-linear dependencies between the HR and RSP signals. Mutual information quantifies the amount of information that one random variable contains about another random variable [28] and captures both linear and non-linear relationships. The MI was calculated using the joint probability distribution of the signals, derived from their joint histogram, and the marginal probabilities for each signal. As mentioned above, these analyses were performed on the z-score signals to ensure consistent results.

**Figure 3:**
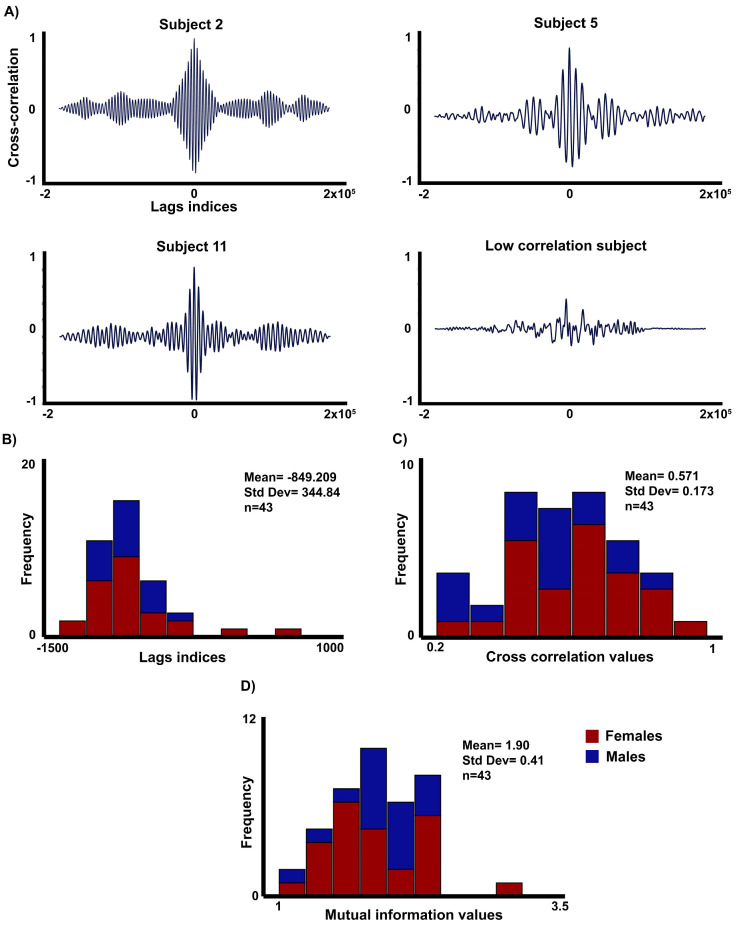
Cross-correlation and mutual information analysis. (**A**) Cross-correlation analysis between the RSP and HR signals in three subjects with the highest correlation coefficients (0.817–0.93) and one subject with low correlation (0.218). (**B**) Histogram with the time-lags indices excluding the subjects with higher time-lag values and very poor cross-correlation. (**C**) Histogram of the cross-correlation values without excluding subjects. (**D**) Histogram with the mutual information values without excluding subjects.

To test for a possible dependence of the continuous HR signal on the continuous RSP signal and its first time derivative, the following continuous models were tested by regression analysis in each subject on the continuous RSP signals and (as shown in Figure 2 and Figure 3) using the Matlab function fitlm.

Continuous model 1 (c-model1):

(1) HRm(t)=b0+b1∗RSP(t)

Continuous model 2 (c-model2) (based on Kapidžić et al., 2014 [13]):

(2) HRm(t)=b0+b1∗RSP(t)+b2∗dRSP/dt

HRm(t): Modelled continuous heart rate signal from the respiratory signal.
RSP (t): Continuous respiratory signal.
dRSP/dt: First-time derivative of RSP(t).

Then, the best model was calculated by the Akaike Information Criterion (AIC) subject by subject [29]. The operationalized variables were the empirical variables RSP and HR (both in z-score), and the computed variables HRm(t), and the first-time derivative of RSP (dRSP/dt). The time-lag values of RSP and HR were obtained by cross-correlation. Statistical analyses were performed in Matlab R2019b (MathWorks) and the Statistical Package for the Social Sciences 26 (SPSS). A simulation, described in the next section, was built up to show that the computation of the RSP time derivative would be able to modulate the time lag between RSP and HR.

## 3. Results

First, we were interested in how the RSP and HR cycles would be related to a possible delay between the RSP and the HR cycles, to be determined by cross-correlation between these two signals. For the cross-correlation analysis (Figure 3), the general results showed negative time-lag indices and high peak correlation when the RSP signal was cross-correlated with HR. There were no significant gender differences in the time-lag and cross-correlation analysis. However, in two subjects the lag indices were extremely large and showed a low correlation peak. These two outliers were detected by the isoutlier function. Excluding these two subjects, the mean value of the lag indices was 849.21 ± SD 344.84, indicating a delayed response for the HR compared to the RSP signal. Similarly, taking into account the relevance of the non-linearities in the study of the influence of the RSP in HR, an MI analysis was added, finding mean values of 1.90 ± SD 0.41. The histogram shows that the MI values are clustered around a moderate level of shared information between HR and RSP signals. No significant effect of gender was found for this metric. To ensure that the linear approach captures the most relevant information about the relationship of the RSP and HR at rest, a correlation analysis was calculated between these two metrics, which showed a significant correlation between MI and cross-correlation (R = 0.498; *p* < 0.001). These results suggest that at rest, most of the common information between HR and RSP signals is captured by linear dependencies.

**Figure 4:**
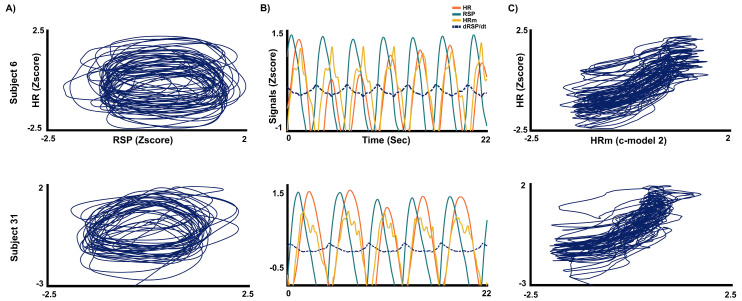
RSP vs. HR (and HRm). (**A**) Closed trajectory of the respiratory signal (RSP) vs. the heart rate signal (HR). (**B**) This figure shows the trend to linearization between the predicted HRm and HR signal when the c-model2, which includes a term of the first-time derivative of RSP, is applied. Please observe the high overlapping between the predicted HRm values from cmodel-2 and the HR signal (in orange). (**C**) When the predicted values of c-model2 (HRm) are represented vs. HR, a linearity of the relationship emerges.

**Figure 5:**
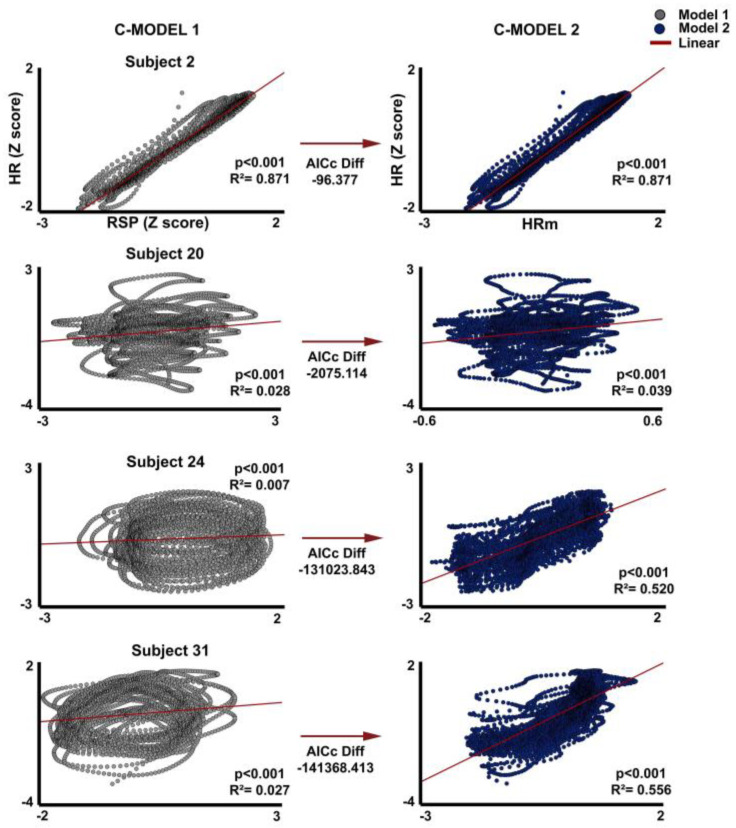
Linear regression of representative subjects. Graphical representation of the linear regressions of HR vs. RSP (left side) and HR vs. the predicted values of c-model2 (HRm). Two subjects with a low AICc difference and two subjects with a high AICc difference are displayed. The same analysis for the complete sample (45 subjects) is displayed in Supplementary Figures S1 and S2. AICc: Akaike information criterion corrected.

To test the dynamic relationship between RSP and HR signals, the continuous models described in Section 2 were applied and tested (c-model1 and c-model2). Note that c-model1 is similar to relating the empirical HR to the empirical RSP, as HRm in c-model1 is linearly related to RSP. Figure 4A shows a pattern similar to a closed trajectory for the relationship of RSP to HR. This closed trajectory is suggested in Figure 4B to be due to the time-lag between RSP and HR. However, when the predicted signal HRm generated by the c-model2 is compared with HR, the phase-lag with HR disappears (Figure 4B orange lines), and a trend showing the linearization of the relationship between the predicted HRm values of c-model2 and empirical HR appears (Figure 4C and Figure 5). The results for all subjects are shown in Supplementary Table S1 and Supplementary Figures S1 and S2. The most explanatory model according to the AICc criterion is c-model2 when compared to c-model1 (45/45 cases). The slope of the first term (proportional to the RSP signal) shows a preponderance of positive slopes (32/45), while the second term (proportional to the first-time derivative of the RSP) shows a preponderance of negative slopes: 44/45. When the total sample of subjects is plotted for the linear regressions between HR vs. RSP, and HR vs. predicted values from c-model2 (HR vs. HRm), the slope of the c-model2 (HRm vs. HR) was always positive and steeper than in c-model1 (HR vs. RSP) (Supplementary Figures S1 and S2).

**Figure 6:**
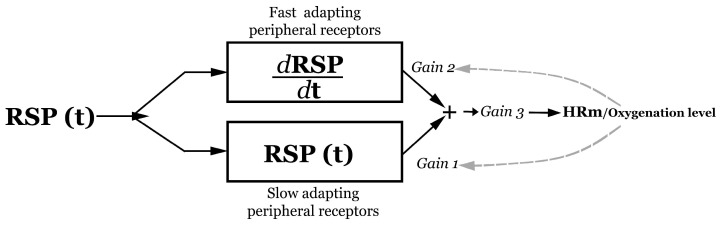
Model of the relationship between activity in the peripheral receptors and the heart rate response, following cmodel-2. RSP: continuous respiratory signal (Recorded_RSP in the script code), HRm: modelled heart rate (Advanced_HR in the script code).

**Figure 7:**
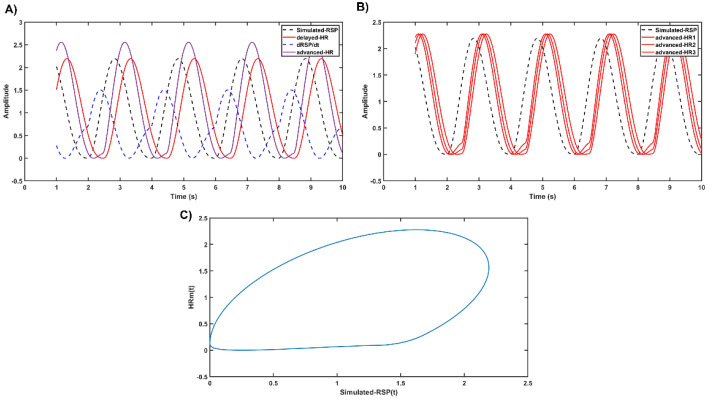
Simulation of heart rate from the respiration signal. (**A**) The simulated RSP(t), its time derivative, and delayed and advanced simulated HRm by c-model2 are displayed. (**B**) The modulations of the time-lags between the simulated RSP(t) and HRm for three different values of the gain2 are displayed. (**C**) Closed trajectory between the simulated RSP(t) and HRm replicating the relationship between the experimentally recorded RSP and HR.

These results suggest that the consideration of dRSP/dt improves the prediction of the HR signal. A possible model for the relationship between peripheral receptor activity and the HR response is shown in Figure 6. To test the hypothesis that the derivative term would have the effect of reducing the delay between RSP and HR, the following simulation (script in the Supplementary Material) is sketched in the diagram of Figure 6. First, (i) a simulated RSP signal (RSP(t)) is generated from an asymmetric and smoothed triangular signal (Simulated_RSP variable in the script’s code) (Figure 7A), (ii) and then a delayed HRm signal is obtained by inserting a delay time into the RSP (t) (delayed_HR in the code), (iii) then dRSP/dt (deriv_RSP in the code) is calculated, which allows (iv) the HRm variable (advanced_HR in the code) to be obtained as a direct application of c-model2 by summing the dRSP/dt term modulated by gain2 to the RSP(t). HRm (advanced_HR) would simulate the empirically recorded HR. By changing the gain2 of the dRSP/dt, it is possible to adjust the delay between the RSP(t) signal (Simulated_RSP in the code) and the HRm (advanced_HR in the code) (Figure 7B). Alternatively, the time-lag can be modulated by changing the gain1 of the RSP(t). Not only the time-lag but also the amplitude of HRm can be modulated by a multiplicative gain3 factor. The dashed line in Figure 6 suggests the possibility, not included in the model, that chemoreceptors would modulate the values of gain1 and/or gain2 values to optimize respiratory gas exchange at the pulmonary alveolar system.

Note that the delay between RSP(t) and HRm included in the model is a consequence of the whole process. By modulating the gain1 for the RSP(t) term, gain2 for the dRSP/dt, or both, it is possible to balance the time-lag between RSP(t) and HRm. The script in the Supplementary Material allows to demonstrate how the adjustment of the gain 1 and/or 2 allows to modulate the time-lag between RSP and HR.

Figure 7A shows the computation of the simulated HRm as a delayed linear relationship of the RSP(t) signal. By adding the derivative of respiration (dRSP/dt) to the RSP(t), the delay of the heart rate with respect to the RSP(t) can be reduced, resulting in the advanced HRm, which is intended to simulate the empirically recorded HR. By changing the gain2 of the dRSP/dt term, the time-lag between the RSP(t) and HRm signals can be modulated (Figure 7B). The amplitude of the HRm signal can also be modulated (gain3). Finally, the closed trajectory observed in most of the subjects for the RSP vs. HR signals, as shown in Figure 4A and Supplementary Figures S1 and S2, is obtained by plotting the simulated RSP(t) vs. the computed HRm (Figure 7C). The physiological feasibility and possible adaptive value of the model will be discussed. Similar results as in Figure 7A–C can be obtained by modulating gain1 (see script in the Supplementary Material).

## 4. Discussion

The dynamic relationship between the RSP signal and the HR signal is supported by the significant cross-correlation, mutual information, and time-lag delay between these two signals. The c-model2 suggests that the amplitude of the heartbeat frequency during RSA would be related to the amplitude of the RSP signal and its first-order time derivative of the RSP amplitude. The inclusion of the time derivative of the RSP allows linearization of the modeling of the cardiopulmonary relationship. This would allow anticipating the state of the lung (inflation and deflation), reducing the delay between the activation of the stretch receptors and the cardiac response, and facilitating gas exchange in the alveolar system, as previously suggested [22,30]. The proposed model has been simulated and allows to confirm that the modulation of the gain of the RSP(t) and/or the dRSP/dt term allows to regulate the time-lag between RSP and HR.

The inclusion of MI analysis in the present report provided additional insights into the nonlinear dynamics of the dynamic relationship between HR and RSP at rest. The results showed values around a moderate level of shared information between HR and RSP signals, similar to the cross-correlation results, and supporting the existing literature on their interconnection [6,12,16,22,31]. Furthermore, the significant correlation between MI and cross-correlation suggests that linear dependencies account for a substantial proportion of the shared information between HR and RSP signals under spontaneous resting state conditions. This finding reinforces the utility of linear approaches in capturing the dynamics of both signals at rest while highlighting the complementary role of non-linear metrics such as MI in exploring additional features in the relationship between these physiological signals. While non-linear analyses provide valuable complementary information, the strong performance of linear metrics suggests that much of the shared information between these signals could be attributed to stable, linear interactions when analyzing young healthy subjects under spontaneous resting state conditions. Possibly, nonlinearities would be more relevant for explaining RSP–HR interactions under stressful, pathological, and aging conditions.

The cross-correlation results show that the RSP signal precedes the HR, the latter with a mean delay of 849.21 ms, with oscillations that are coupled in most subjects, according to the concept of the RSA of HR causally linked to RSP. These results support the well-known hypothesis that respiratory cycles influence the cardiac response [16,31]. The observed time-lag values are consistent with some previous measurements, for instance, Saul et al. [32] proposed that R–R interval changes follow breathing with a time lag of about 0.3 s. The presence of a delay between the respiratory and cardiac cycles [33] does not necessarily resolve the controversy about a central or peripheral mechanism for generating RSA [24], since different modulatory pathways with synchronized but different time delays would explain the respiratory-to-cardiac cycle time-lag. This result is consistent with proposals on the controlling role of respiration over cardiac cycles by central [34]; or peripheral mechanisms linked to respiratory [19], or respiratory-related signals [14,21,22]. Therefore, a time-lag delay between the respiratory and cardiac cycles should be included in the dynamic modeling of RSA.

The relationship between both signals is more evident when considering the maximally explanatory model for HR from RSP (c-model2), in which the inclusion of the first-order time derivative of the respiratory signal improves the ability of RSP to predict HR. A result, previously obtained for the inspiratory phase, and incorporating the RSP and its time derivative, is used to predict HR [13]. From a modeling perspective, the first-order time derivative allows a transition from a closed trajectory for the relationship between RSP and. HR to a more linear one. The dependence of the RSP signal on HR has previously been observed and quantified using autoregressive moving average techniques [31], demonstrating the influence of the time evolution of RSP (and arterial pressure) on HR. However, such modeling does not establish a precise relationship between the time derivative of RSP and HR, a relationship that has been previously described [13], suggesting possible mechanisms for pathological conditions. A potential advantage of the c-model2 is that it suggests the presence of an anticipatory computation of the respiratory phase by partially discounting the time delay between pulmonary stretch receptors activation and HR response. This could optimize gas exchange [22,30] in the alveolar pulmonary system between RSP and HR by a precise time-lag between both signals.

An important point to note about c-model2 is that when the predicted values of HR from c-model2 (HRm) are regressed with HR, a linearization with a positive slope appears. This result should be interpreted from a modeling perspective as if RSP, once compensated by the rate of change (second term of c-model2), linearly drives the cardiac cycle during the resting state, supporting the view of a modulation of HR from the respiratory cycle [7,8]. One suggestion would be that slowly adapting stretch receptors would carry the first term of c-model2 (proportional to the RSP signal) and the fast-adapting receptors, which are more sensitive to changes in RSP, would carry the second term of c-model2, proportional to the time derivative of RSP. The presence of rapid and fast adapting stretch receptors in the respiratory muscles would carry the RSP and dRSP/dt signals to central vagal centers [19] without discarding the possible role of arterial pressure mechanoreceptors in the carotid body. The possible role of stretch receptors must be considered tentatively, but would explain the influence of the RSP time derivative on HR. Of course, the cardiac response has much broader peripheral and central regulatory points, including the carotid body receptors, which are difficult to assess from the present results. However, the reported simulation must be understood in terms of signal processing modeling rather than as a specific physiological proposal that would require pharmacological or physiological intervention and/or the recording of peripheral and central nervous system generators.

The possibility of obtaining a precise time-lag between RSP and HR during RSA, by regulating the gain of RSP or dRSP/dt, is important to optimize the synchrony between the inspiration phase at the alveolar level and the arrival of an optimal amount of deoxygenated blood at the alveolar level. This is a dynamically complex problem to be computed due to the physics of the gases and fluids passing through the bronchi and bronchioles and the different ramifications at the cardiovascular level and blood flow through them. The present model and simulation allow the possibility of regulating the time-lag by modifying the proportion of RSP and dRSP/dt signal at the neural level. However, modeling neural processing is also complex. To model the exact relationship between the contribution of the RSP and dRSP/dt to HR would require a lot of physiological information, taking into account the mechanics of the respiratory system to activate the stretch receptors, the activation of the receptors, the central processing, the activation of the heart, and the heart electrical transmission. Such an amount of physiological information would be impracticable in humans. However, the proposed model opens the possibility of finding the most adaptive and energetically efficient oxygenation level by regulating the cardiorespiratory time-lag during RSA, with chemoreceptors providing information about the efficacy of gas exchange [21,22,23]. Assessment of blood oxygenation levels would hypothetically induce changes in the model gain1 to optimize the synchrony between atmospheric gas pressure and hemoglobin concentration at the alveolus to optimize gas exchange [22,30]. But also, the possibility of modulating the delay between RSP and HR could be modulated by precise timing initiation of the inspiratory phase from the cardiac activity [35].

The present report makes some suggestions about the possible control of HR by peripheral stretch receptors related to the RSP cycle. However, to confirm this approach, the forced control of the RSP cycle, pharmacological approaches, and/or invasive recordings of the peripheral and central nervous system would be required to follow the neural feedback chain, central generators, and autonomic cardiac control. Also, testing in pathological conditions where the interaction between RSP and HR is disturbed would be very useful to understand this relationship.

## 5. Conclusions

In conclusion, to model the possible inter-relationship between the RSP and HR signals, a time-lag delay between RSP and HR, which could be reduced by taking into account the time derivative of the RSP signal, would give a good account of the RSP–HR cardiorespiratory dynamics during the resting state in young healthy subjects. The proposed model allows the linearization of the relationship between RSP and HR and, by modulating the gains of the RSP and/or dRSP/dt terms, allows the adjustment of the time-lag between RSP and HR, possibly allowing the optimization of the gas exchange between the vascular and pulmonary systems.

## Data Availability

The data presented in this study are openly available on FigShare at https://doi.org/10.6084/m9.figshare.27332226.v1.

## Supplementary Materials

The following supporting information can be downloaded at: https://www.mdpi.com/article/10.3390/e26121083/s1. Table S1. Linear model regression of continuous waves of HR and RSP z-score signals including the time derivatives of RSP. Figure S1. Linear regression of the continuous signal. Graphical representation of the linear regressions of HR vs. RSP (left side) and HR vs. the predicted values of c-model2 for the first 24 Subjects (1–24 Subjects). Figure S2. Linear regression of the continuous signal. Graphical representation of the linear regressions of HR vs. RSP (left side) and HR vs. the predicted values of c-model2 for the last 21 Subjects (25–45 Subjects). Supplementary code. Matlab custom script to perform a simulation that allows to replicate the physiological relationship between RSP and HR.

## References

[1] Plaxton W., Storey K.B. (2004). Principles of metabolic control. Functional Metabolism: Regulation and Adaptation.

[2] Cannon W.B. (1932). The Wisdom of the Body.

[3] Zholtkevych G.N., Nosov K.V., Bespalov Y.G., Rak L.I., Abhishek M., Vysotskaya E.V. (2018). Descriptive Modeling of the Dynamical Systems and Determination of Feedback Homeostasis at Different Levels of Life Organization. Acta Biotheor..

[4] McClintock C., Stefanovska A., Stefanovska A., McClintock C. (2021). Chapter 1: Introduction. Physics of Biological Oscillators: New Insights into Non-Equilibrium and Non-Autonomous Systems.

[5] Nitzan M., de Boer H., Turivnenko S., Babchenko A., Sapoznikov D. (1994). Power spectrum analysis of spontaneous fluctuations in the photoplethysmographic signal. J. Basic Clin. Physiol. Pharmacol..

[6] Schäfer C., Rosenblum M.G., Kurths J., Abel H.H. (1998). Heartbeat synchronized with ventilation. Nature.

[7] Cairo B., de Abreu R.M., Bari V., Gelpi F., De Maria B., Rehder-Santos P., Sakaguchi C.A., da Silva C.D., De Favari Signini É., Catai A.M. (2021). Optimizing phase variability threshold for automated synchrogram analysis of cardiorespiratory interactions in amateur cyclists. Philos. Trans. A Math. Phys. Eng. Sci..

[8] Yasuma F., Hayano J. (2001). Augmentation of respiratory sinus arrhythmia in response to progressive hypercapnia in conscious dogs. Am. J. Physiol. Heart Circ. Physiol..

[9] Zhang Q., Patwardhan A.R., Knapp C.F., Evans J.M. (2015). Cardiovascular and cardiorespiratory phase synchronization in normovolemic and hypovolemic humans. Eur. J. Appl. Physiol..

[10] Porta A., Faes L., Bari V., Marchi A., Bassani T., Nollo G., Perseguini N.M., Milan J., Minatel V., Borghi-Silva A. (2014). Effect of age on complexity and causality of the cardiovascular control: Comparison between model-based and model-free approaches. PLoS ONE.

[11] de Abreu R.M., Catai A.M., Cairo B., Rehder-Santos P., Silva C.D., Signini É.D., Sakaguchi C.A., Porta A. (2020). A Transfer Entropy Approach for the Assessment of the Impact of Inspiratory Muscle Training on the Cardiorespiratory Coupling of Amateur Cyclists. Front. Physiol..

[12] de Abreu R.M., Cairo B., Porta A. (2023). On the significance of estimating cardiorespiratory coupling strength in sports medicine. Front. Netw. Physiol..

[13] Kapidžić A., Platiša M.M., Bojić T., Kalauzi A. (2014). RR interval-respiratory signal waveform modeling in human slow paced and spontaneous breathing. Respir. Physiol. Neurobiol..

[14] Bernardi L., Porta C., Gabutti A., Spicuzza L., Sleight P. (2001). Modulatory effects of respiration. Auton. Neurosci..

[15] Spyer K.M. (1994). Annual review prize lecture. Central nervous mechanisms contributing to cardiovascular control. J. Physiol..

[16] Feldman J.L., McCrimmon D.R., Morrison S.F. (2013). Neural Control of Respiratory and Cardiovascular Functions. Fundamental Neuroscience.

[17] Eckberg D.L. (2003). The human respiratory gate. J. Physiol..

[18] Grossman P., Taylor E.W. (2007). Toward understanding respiratory sinus arrhythmia: Relations to cardiac vagal tone, evolution and biobehavioral functions. Biol. Psychol..

[19] Russo M.A., Santarelli D.M., O’Rourke D. (2017). The physiological effects of slow breathing in the healthy human. Breathe.

[20] Bernardi L., Valle F., Leuzzi S., Rinaldi M., Marchesi E., Falcone C., Martinelli L., Viganó M., Finardi G., Radaelli A. (1994). Non-respiratory components of heart rate variability in heart transplant recipients: Evidence of autonomic reinnervation?. Clin. Sci..

[21] Tafil-Klawe M., Trzebski A., Klawe J. (1985). Contribution of the carotid chemoreceptor reflex to the mechanism of respiratory sinus arrhythmia in young healthy and hypertensive humans. Acta Physiol. Pol..

[22] Yasuma F., Hayano J. (2004). Respiratory sinus arrhythmia: Why does the heartbeat synchronize with respiratory rhythm?. Chest.

[23] López-Barneo J., Ortega-Sáenz P., González-Rodríguez P., Fernández-Agüera M.C., Macías D., Pardal R., Gao L. (2016). Oxygen-sensing by arterial chemoreceptors: Mechanisms and medical translation. Mol. Aspects. Med..

[24] Karemaker J.M. (2009). Counterpoint: Respiratory sinus arrhythmia is due to the baroreflex mechanism. J. Appl. Physiol..

[25] Riedl M., Müller A., Kraemer J.F., Penzel T., Kurths J., Wessel N. (2014). Cardio-respiratory coordination increases during sleep apnea. PLoS ONE.

[26] Krause H., Kraemer J.F., Penzel T., Kurths J., Wessel N. (2017). On the difference of cardiorespiratory synchronisation and coordination. Chaos.

[27] Hayano J., Iwase S., Hayano J., Orimo S. (2017). Introduction to heart rate variability. Clinical Assessment of the Autonomic Nervous System.

[28] Cover T.M., Thomas J.A., Cover T.M., Thomas J.A. (2005). Entropy, Relative Entropy, and Mutual Information. Elements of Information Theory.

[29] Akaike H. (1974). A new look at the statistical model identification. IEEE Trans. Autom. Control.

[30] Hayano J., Yasuma F. (2003). Hypothesis: Respiratory sinus arrhythmia is an intrinsic resting function of cardiopulmonary system. Cardiovasc. Res..

[31] Triedman J.K., Perrott M.H., Cohen R.J., Saul J.P. (1995). Respiratory sinus arrhythmia: Time domain characterization using autoregressive moving average analysis. Am. J. Physiol..

[32] Saul J.P., Berger R.D., Chen M.H., Cohen R.J. (1989). Transfer function analysis of autonomic regulation. II. Respiratory sinus arrhythmia. Am. J. Physiol..

[33] Lewis G.F., Furman S.A., McCool M.F., Porges S.W. (2012). Statistical strategies to quantify respiratory sinus arrhythmia: Are commonly used metrics equivalent?. Biol. Psychol..

[34] Spyer K.M., Lambert J.H., Thomas T. (1997). Central nervous system control of cardiovascular function: Neural mechanisms and novel modulators. Clin. Exp. Pharmacol. Physiol..

[35] Tzeng Y.C., Larsen P.D., Galletly D.C. (2007). Mechanism of cardioventilatory coupling: Insights from cardiac pacing, vagotomy, and sinoaortic denervation in the anesthetized rat. Am. J. Physiol. Heart Circ. Physiol..

