# Supplementary material for: Predictive Modeling of Heart Rate from Respiratory Signals at Rest in Young Healthy Humans
Source: Entropy (Basel). 2024 Dec 11;26(12):1083. doi: 10.3390/e26121083 (PMC11675163; doi:10.3390/e26121083)
Supplement: Supplementary file 1 [file entropy-26-01083-s001.zip › entropy-3315165-supplementary.pdf]

SUPPLEMENTARY MATERIAL

Supplementary Table S1.

Linear Model regression of continuous waves of HR and RSP z-score signals including the time derivatives of RSP

**S**	**G**	**C-MODEL 1**			**C-MODEL 2**						**AICc criterion difference**
					**First term**		**Second term**		**Model**		
		R^2	*B sign*	*p*	*B sign*	*p*	*B sign*	*p*	*R^2*	*p*	
1	M	0.499	+	<0.001	+	<0.001	-	<0.001	0.536	<0.001	-14016.690
2	F	0.871	+	<0.001	+	<0.001	-	<0.001	0.871	<0.001	-96.377
3	F	0.009	+	<0.001	+	<0.001	-	<0.001	0.107	<0.001	-18599.860
4	F	0.169	+	<0.001	+	<0.001	-	<0.001	0.206	<0.001	-8231.609
5	F	0.510	+	<0.001	+	<0.001	-	<0.001	0.656	<0.001	-63740.620
6	M	0.003	+	<0.001	+	<0.001	-	<0.001	0.505	<0.001	-126100.695
7	F	0.069	+	<0.001	+	<0.001	-	<0.001	0.215	<0.001	-30663.759
8	F	0.011	-	<0.001	-	<0.001	-	<0.001	0.130	<0.001	-23041.101
9	M	0.0002	-	<0.001	-	<0.001	-	<0.001	0.016	<0.001	-2873.193
10	F	0.014	+	<0.001	+	<0.001	-	<0.001	0.214	<0.001	-40664.214
11	F	0.189	+	<0.001	+	<0.001	-	<0.001	0.538	<0.001	-101229.832
12	M	0.084	+	<0.001	+	<0.001	-	<0.001	0.144	<0.001	-12125.522
13	M	0.151	+	<0.001	+	<0.001	-	<0.001	0.242	<0.001	-20427.410
14	M	0.002	-	<0.001	-	<0.001	-	<0.001	0.106	<0.001	-19783.778
15	F	0.0009	+	<0.001	+	<0.001	-	<0.001	0.253	<0.001	-52412.418
16	M	0.0008	+	<0.001	+	<0.001	-	<0.001	0.057	<0.001	-10349.750
17	F	0.137	-	<0.001	-	<0.001	-	<0.001	0.522	<0.001	-106424.527
18	F	0.494	+	<0.001	+	<0.001	+	<0.001	0.524	<0.001	-10880.178
19	F	0.019	-	<0.001	-	<0.001	-	<0.001	0.221	<0.001	-41571.204
20	M	0.028	+	<0.001	+	<0.001	-	<0.001	0.039	<0.001	-2075.114
21	F	0.0009	+	<0.001	+	<0.001	-	<0.001	0.418	<0.001	-97208.993
22	M	0.003	-	<0.001	-	<0.001	-	<0.001	0.160	<0.001	-30942.220
23	F	0.057	+	<0.001	+	<0.001	-	<0.001	0.313	<0.001	-56886.351
24	F	0.007	+	<0.001	+	<0.001	-	<0.001	0.520	<0.001	-131023.843
25	F	0.079	-	<0.001	-	<0.001	-	<0.001	0.156	<0.001	-15733.695
26	M	0.039	+	<0.001	+	<0.001	-	<0.001	0.217	<0.001	-36932.275
27	M	0.019	+	<0.001	+	<0.001	-	<0.001	0.076	<0.001	-10827.617
28	F	0.021	-	<0.001	-	<0.001	-	<0.001	0.240	<0.001	-45591.367
29	M	0.371	+	<0.001	+	<0.001	-	<0.001	0.592	<0.001	-77610.990
30	F	0.096	+	<0.001	+	<0.001	-	<0.001	0.253	<0.001	-34240.525
31	F	0.027	+	<0.001	+	<0.001	-	<0.001	0.556	<0.001	-141368.413
32	F	0.349	+	<0.001	+	<0.001	-	<0.001	0.446	<0.001	-28865.138
33	F	0.006	-	<0.001	-	<0.001	-	<0.001	0.172	<0.001	-32795.927
34	M	0.070	-	<0.001	-	<0.001	-	<0.001	0.293	<0.001	-49279.915
35	F	0.005	-	<0.001	-	<0.001	-	<0.001	0.144	<0.001	-27137.253
36	F	0.003	-	<0.001	-	<0.001	-	<0.001	0.252	<0.001	-51665.260
37	M	0.056	+	<0.001	+	<0.001	-	<0.001	0.185	<0.001	-26494.610
38	F	0.020	-	<0.001	-	<0.001	-	<0.001	0.075	<0.001	-10430.486
39	F	0.021	+	<0.001	+	<0.001	-	<0.001	0.278	<0.001	-54828.708
40	F	0.467	+	<0.001	+	<0.001	-	<0.001	0.566	<0.001	-37071.713
41	F	0.053	+	<0.001	+	<0.001	-	<0.001	0.233	<0.001	-37894.951
42	M	0.005	+	<0.001	+	<0.001	-	<0.001	0.053	<0.001	-8801.937
43	M	0.164	+	<0.001	+	<0.001	-	<0.001	0.371	<0.001	-51320.635
44	M	0.0002	+	<0.001	+	<0.001	-	<0.001	0.363	<0.001	-81056.524
45	M	0.033	+	<0.001	+	<0.001	-	<0.001	0.211	<0.001	-36617.407

Abbreviations: S: Subjects; G: Gender; M: Male; F: Female. B sign: Beta sign. AICc: Akaike Information Criterion.

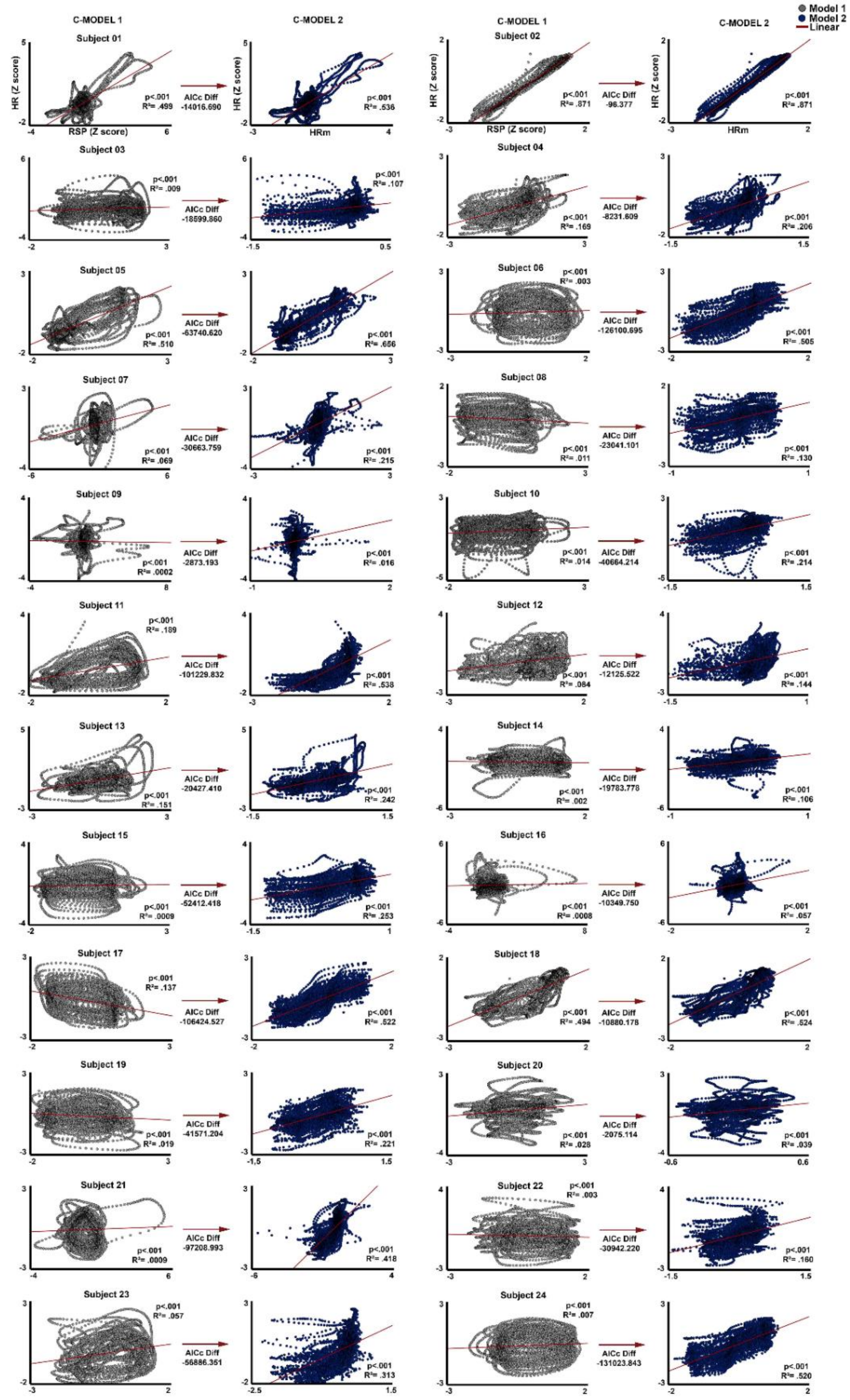

Supplementary Figure S1. Linear Regression of the Continuous Signal. Graphical representation of the linear regressions of HR vs RSP (left side) and HR vs the predicted values of c-model2 for the first 24 Subjects (1-24Subjects).

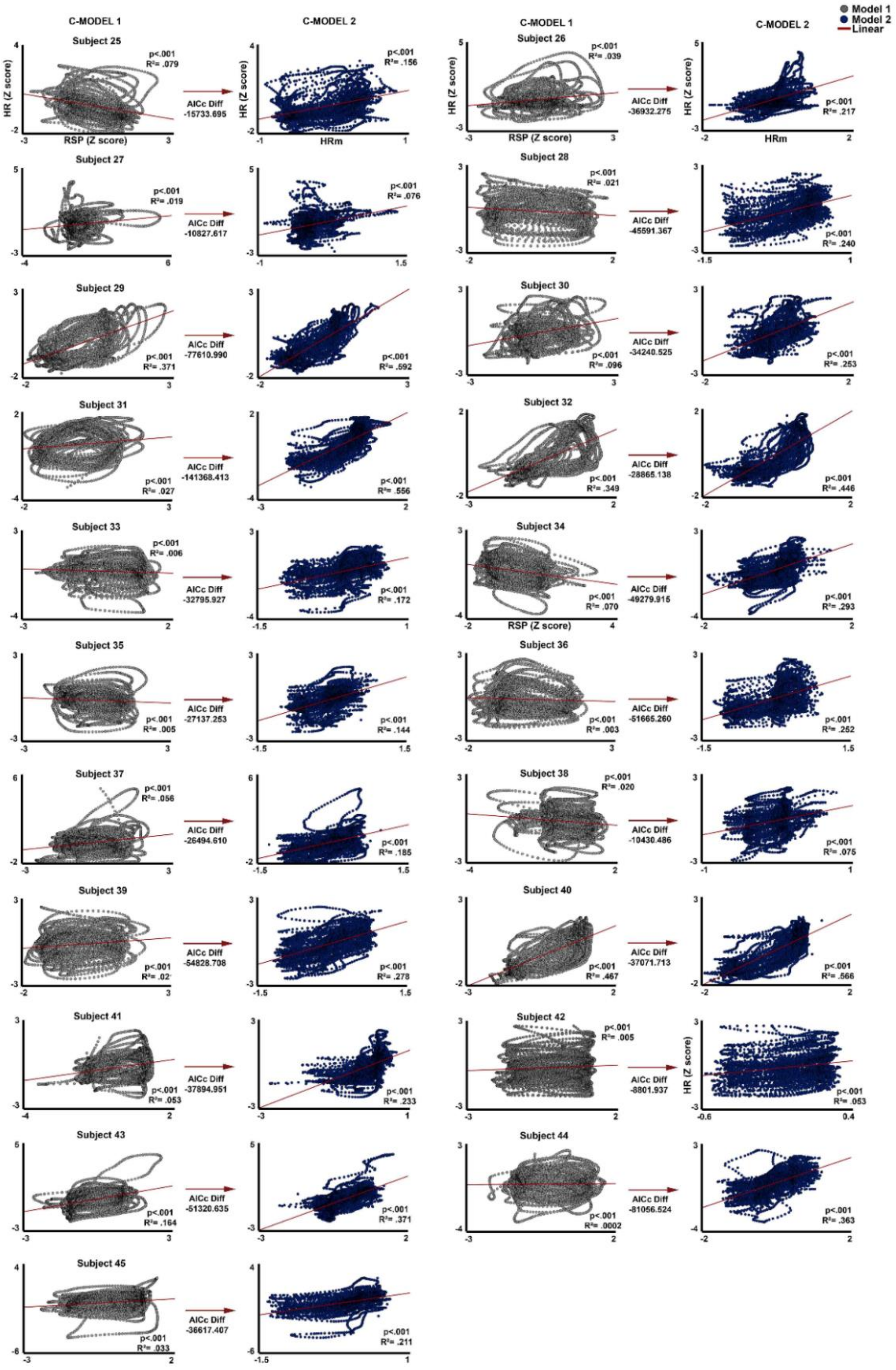

Supplementary Figure S2. Linear Regression of the Continuous Signal. Graphical representation of the linear regressions of HR vs RSP (left side) and HR vs the predicted values of c-model2 for the last 21 Subjects (25-45 Subjects).

Supplementary code. Matlab custom script to perform a simulation that allows to replicate the physiological relationship between RSP and HR.

```
clear all
close all
% Parameters to create the respiratory wave
amplitude = 1; % Amplitude of the triangular wave
period = 2; % Period of the triangular wave (in seconds)
duty_cycle = 0.01; % Duty cycle (portion of the period for the rising edge)
duration = 10; % Total duration of the signal (in seconds)
fs = 1000; % Sampling frequency (in Hz)
smoothing_window = 100; % Sizfor i=1:1:180000e of the moving average smoothing window
delay_time = 0.5; % Delay in seconds
% Time vector
t = 0:1/fs:duration;
% Generate the asymmetric triangular wave multiplied by a sinus wave to simulate respirati
on
tri_wave_2 = amplitude * sawtooth(2 * pi * (1/period) * t, duty_cycle);
a=min(tri_wave_2);
tri_wave=tri_wave_2+abs(a);
seno_2=amplitude * sin(((2 * pi * (1/period) * t))-(pi/2));
b=min(seno_2);
seno=seno_2+abs(b);
c=tri_wave .* seno;

% Apply smoothing using a moving average filter

Simulated_RSP = smoothdata(c, 'movmean', smoothing_window);

% Create delayed version of the smoothed signal to represent a HR signal
% controlled by respiration plus a delay due to stretch receptors, synaptic and neural con
duction
%and heart electrical conduction and contraction delays
delay_samples = round(delay_time * fs); % Convert delay time to samples
delayed_HR = [zeros(1, delay_samples), Simulated_RSP(1:end-delay_samples)];

figure
plot(t((delay_samples*2):end), Simulated_RSP((delay_samples*2):end),'k--');
xlabel('Time (s)');
ylabel('Amplitude');
hold on
plot(t((delay_samples*2):end), delayed_HR((delay_samples*2):end),'r' );


% compute the derivative of RSP

deriv=diff (Simulated_RSP);
normalization=200;
derivv=(normalization*deriv);
a=min(derivv((delay_samples+1):end));
derivv=derivv+abs(a);
deriv_RSP=derivv;%smoothdata(derivv, 'movmean', smoothing_window*8);
delayed_HR=delayed_HR(1:end-1);
Simulated_RSP=Simulated_RSP(1:end-1);
t=t(1:end-1);
hold on
plot(t((delay_samples*2):end), deriv_RSP((delay_samples*2):end),'b--');
```

```
% Compute advanced_HR as the combination of the RSP and its derivative
%advanced_HR= Simulated_RSP+deriv_RSP;


gain1=1;
gain2=1;
advanced_HR=(gain1 * Simulated_RSP) + (gain2 * deriv_RSP);
advanced_HR = [zeros(1, delay_samples), advanced_HR(1:end-delay_samples)];
d=min(advanced_HR(2000:6000));
advanced_HR=advanced_HR-abs(d);

plot(t((delay_samples*2):end), advanced_HR((delay_samples*2):end));
legend('Simulated-RSP','delayed-HR', 'dRSP/dt','advanced-HR');
title('Simulation fig-1: Simulation Recorded RSP, Delayed and advanced HR');
% Another possibility corresponds to he possibility of regulating the
% time-lag
% between the respiration and heart rate during sinus arrhytmia, to optimize gas exchange,
  by changing
% the gain of derivative of RSP
% The summing  of RSP + dRSP/dt could occur at the cardiovascular
% centers in the brainstem
gain1=1;
gain2=0.5;
advanced_HR_1= (gain1 * Simulated_RSP)+(deriv_RSP *gain2);
advanced_HR_1 = [zeros(1, delay_samples), advanced_HR_1(1:end-delay_samples)];
d=min(advanced_HR_1(2000:6000));
advanced_HR_1=advanced_HR_1-abs(d);

gain1=1;
gain2=1;
advanced_HR_2= (gain1 * Simulated_RSP)+(deriv_RSP *gain2);
advanced_HR_2 = [zeros(1, delay_samples), advanced_HR_2(1:end-delay_samples)];
d=min(advanced_HR_2(2000:6000));
advanced_HR_2=advanced_HR_2-abs(d);

gain1=1;
gain2=1.5;
advanced_HR_3= (gain1 * Simulated_RSP)+(deriv_RSP *gain2);
advanced_HR_3 = [zeros(1, delay_samples), advanced_HR_3(1:end-delay_samples)];
d=min(advanced_HR_3(2000:6000));
advanced_HR_3=advanced_HR_3-abs(d);

figure
plot(t((delay_samples*2):end), Simulated_RSP((delay_samples*2):end),'k--');
xlabel('Time (s)');
ylabel('Amplitude');
hold on
plot(t((delay_samples*2):end), advanced_HR_1((delay_samples*2):end),'r' );
plot(t((delay_samples*2):end), advanced_HR_2((delay_samples*2):end),'r' );
plot(t((delay_samples*2):end), advanced_HR_3((delay_samples*2):end),'r' );
legend('Simulated-RSP','advanced-HR1', 'advanced-HR2','advanced-HR3');
title(['Simulation fig-2:Change of HR time-lag RSP-HR', ' by modifying dRSP/dt gain2']);

% A final adjustement can be done for obtainng the same amplitude for all
% the different HR signals with different time-lahs between RSP and HR, by means of adjunt
ing the gain 3
% of the model.
```

```
a1=max(advanced_HR_1);
a2=max(advanced_HR_2);
a3=max(advanced_HR_3);

gain3_1=a1/a1;
gain3_2=a1/a2;
gain3_3=a1/a3;
advanced_HR_1=advanced_HR_1 *gain3_1;
advanced_HR_2=advanced_HR_2 *gain3_2;
advanced_HR_3=advanced_HR_3 *gain3_3;

figure
plot(t((delay_samples*2):end), Simulated_RSP((delay_samples*2):end),'k--');
xlabel('Time (s)');
ylabel('Amplitude');
hold on
plot(t((delay_samples*2):end), advanced_HR_1((delay_samples*2):end),'r' );
plot(t((delay_samples*2):end), advanced_HR_2((delay_samples*2):end),'r' );
plot(t((delay_samples*2):end), advanced_HR_3((delay_samples*2):end),'r' );
legend('Simulated-RSP','advanced-HR1', 'advanced-HR2','advanced-HR3');
title(['Simulation fig-3: Change of HR amplitude', ' by modifying  gain3']);

figure
plot (Simulated_RSP((delay_samples*2):end), (advanced_HR_2 ((delay_samples*2):end)));
xlabel('Simulated-RSP(t)');
ylabel('HRm(t)');;
title(['Simulation fig-4:Simulation of the graphs RSP vs HR']);



% Another possibility corresponds to he possibility for regulating the
% time-lag
% between the respiration and heart rate during sinus arrhytmia, to optimize gas exchange,
  by changing
% the gain1  of RSP, and keeping constant the gain2 of dRSP/dt
% The summing  of RSP + dRSP/dt could occur at the cardiovascular
% centers in the brainstem
gain1=0.5;
gain2=1;
advanced_HR_1= (gain1 * Simulated_RSP)+(deriv_RSP *gain2);
advanced_HR_1 = [zeros(1, delay_samples), advanced_HR_1(1:end-delay_samples)];
d=min(advanced_HR_1(2000:6000));
advanced_HR_1=advanced_HR_1-abs(d);

gain1=1;
gain2=1;
advanced_HR_2= (gain1 * Simulated_RSP)+(deriv_RSP *gain2);
advanced_HR_2 = [zeros(1, delay_samples), advanced_HR_2(1:end-delay_samples)];
d=min(advanced_HR_2(2000:6000));
advanced_HR_2=advanced_HR_2-abs(d);

gain1=1.5;
gain2=1;
advanced_HR_3= (gain1 * Simulated_RSP)+(deriv_RSP *gain2);
advanced_HR_3 = [zeros(1, delay_samples), advanced_HR_3(1:end-delay_samples)];
d=min(advanced_HR_3(2000:6000));
advanced_HR_3=advanced_HR_3-abs(d);
```

```
figure
plot(t((delay_samples*2):end), Simulated_RSP((delay_samples*2):end),'k--');
xlabel('Time (s)');
ylabel('Amplitude');
hold on
plot(t((delay_samples*2):end), advanced_HR_1((delay_samples*2):end),'r' );
plot(t((delay_samples*2):end), advanced_HR_2((delay_samples*2):end),'r' );
plot(t((delay_samples*2):end), advanced_HR_3((delay_samples*2):end),'r' );
legend(' Simulated-RSP','advanced-HR1', 'advanced-HR2','advanced-HR3');
title(['Simulation fig-5:Change of time-lag RSP-HR', ' by modifying RSP gain1']);

% A final adjustement can be done for obtainng the same amplitude for all
% the different HR signals with different time-lags between RSP and HR, by means of adjunt
ing the gain 3
% of the model.
a1=max(advanced_HR_1);
a2=max(advanced_HR_2);
a3=max(advanced_HR_3);

gain3_1=a1/a1;
gain3_2=a1/a2;
gain3_3=a1/a3;
advanced_HR_1=advanced_HR_1 *gain3_1;
advanced_HR_2=advanced_HR_2 *gain3_2;
advanced_HR_3=advanced_HR_3 *gain3_3;

figure
plot(t((delay_samples*2):end), Simulated_RSP((delay_samples*2):end),'k--');
xlabel('Time (s)');
ylabel('Amplitude');
hold on
plot(t((delay_samples*2):end), advanced_HR_1((delay_samples*2):end),'r' );
plot(t((delay_samples*2):end), advanced_HR_2((delay_samples*2):end),'r' );
plot(t((delay_samples*2):end), advanced_HR_3((delay_samples*2):end),'r' );
legend('Simulated-RSP','advanced-HR1', 'advanced-HR2','advanced-HR3');
title(['Simulation fig-6:Change of HR amplitude', ' by modifying RSP gain3']);

figure
plot (Simulated_RSP((delay_samples*2):end), (advanced_HR_2 ((delay_samples*2):end)));
xlabel('Simulated-RSP(t)');
ylabel('HRm(t)');;
title(['Simulation fig-7:Simulation of the graphs RSP vs HR']);
```
